# 1,8-Cineole inhibits biofilm formation and bacterial pathogenicity by suppressing *luxS* gene expression in *Escherichia coli*

**DOI:** 10.3389/fphar.2022.988245

**Published:** 2022-10-14

**Authors:** Yiming Wang, Yu Zhang, Xu Song, Chunlin Fang, Rui Xing, Lu Liu, Xinghong Zhao, Yuanfeng Zou, Lixia Li, Renyong Jia, Gang Ye, Fei Shi, Xun Zhou, Yingying Zhang, Hongping Wan, Qin Wei, Zhongqiong Yin

**Affiliations:** ^1^ Natural Medicine Research Center, College of Veterinary Medicine, Sichuan Agricultural University, Chengdu, China; ^2^ Chengdu Agricultural College, Chengdu, China; ^3^ Chengdu QianKun Veterinary Pharmaceutical Co., Ltd., Chengdu, China; ^4^ Key Laboratory of Animal Disease and Human Health of Sichuan Province, Sichuan Agricultural University, Chengdu, China; ^5^ Sichuan Oil Cinnamon Engineering Technology Research Center, Yibin University, Yibin, China

**Keywords:** 1, 8-cineole, quorum sensing, biofilm, *Escherichia coli*, LuxS gene, pathogenicity

## Abstract

In recent years, with frequent reports of multi-drug resistant strains, bacteria antibiotic resistance has become an increasingly serious health problem worldwide. One of the most promising ways for combating bacterial infections and antibiotic resistance is development of quorum-sensing (QS) interfering drugs. In this study, the results show that 1,8-cineole inhibited the expression of QS as well as the virulence genes in *Escherichia coli* O101 *(E. coli* O101*)* with a 65% inhibition rate against *luxS* gene. Therefore, we hypothesized that 1,8-cineole may inhibit the biofilm formation and reduce the pathogenicity of *E. coli* O101 by inhibiting the expression of *luxS* gene. To confirm our hypotheses, a *luxS* gene deleted *E. coli* O101 was constructed. The results show that the biofilm formation, motility, structure and pathogenicity of *E. coli* O101 were significantly inhibited following deletion of the *luxS* gene. In addition, the transcript levels of QS and virulence genes of *E. coli* O101 were also significantly down-regulated. Interestingly, 1,8-cineole no longer had a significant inhibitory effect on the related phenotype and gene expression of *E. coli* O101 without *luxS* gene. In conclusion, the results show that 1,8-cineole can affect bacterial biofilm formation and pathogenicity by suppressing the expression of *luxS* gene in *E. coli* O101, which could provide a new perspective for dealing with the biofilm problem of pathogenic bacteria.

## Introduction

Due to the advantages of low toxicity and low levels of acquired resistance, compounds isolated from plant and their derivatives have played an increasingly important role in anti-infective therapy in recent years (Luo, Dong et al., 2017). Antibacterial activity is one of the most promising effect of this type of compounds (Álvarez-Martínez et al., 2021). Among these promising antimicrobial plant-derived natural compounds, essential oils are vital parts of them. Essential oils are secondary metabolites of aromatic plants with significant antibacterial activity and quorum-sensing (QS) inhibition (Wang W. et al., 2019). The monoterpene 1,8-cineole, the major constituent of eucalyptus species (Juergens, Worth et al., 2020), has shown good anti-inflammatory, analgesic and antioxidant activities (Kennedy and Sperandio. 2016, Yin, Liu et al., 2020).

Bacteria are social organisms able to build complex structures such as biofilms, which are highly organized surface-associated communities of microorganisms encased within a self-produced extracellular matrix (Guzzo, Scognamiglio et al., 2020). Biofilm formation is an important self-defense mechanism of bacteria (Cepas, López et al., 2018). The presence of biofilm reduces the effectiveness of antibiotics and fungicides, resulting in the development of microbial resistance, which are also the major cause for many chronic infections (Koo et al., 2017a). Obviously, antimicrobial therapy targeting bacterial biofilms is a prospective therapeutic strategy (Koo et al., 2017b). It has been shown that the absence of the quorum sensing system will result in loose, poorly adherent, and even structurally incomplete in bacterial biofilms (Kumar, Umesha et al., 2016). (Williams 2002).

QS is the process to realize chemical information exchange between bacteria, which can make the bacterial community change its behavior synchronously to adapt to the changes of the population density and species of neighboring communities (Mukherjee and Bassler 2019a). The QS systems can be generally categorized into four types. The LuxI-LuxR QS system, which is extensively found in Gram-negative bacteria and regulated by Acyl-homoserine lactones (AHLs). The second one is the Gram-positive bacteria QS system, which is regulated by Auto-inducing peptides (AIPs, small molecule signal peptides). The LuxS/AI-2 system is the third type, coexisting in both Gram-negative and Gram-positive bacteria (Wang Y. et al., 2019), which can affect the growth characteristics, biofilm formation, virulence, and metabolism of bacteria (Zhang, Ku et al., 2019). The last one is AI-3/epinephrine/norepinephrine QS system, which also can regulate a variety of physiological activities, such as biofilm formation, spore production, virulence and the transmission of information (Kendall and Sperandio 2007).

AI-2 is the signaling molecule in the LuxS/AI-2 QS system, which can modulate gene expression and consequently impact microbial behavior, and it is the most common kind of microbial regulation (Wang, Liu et al., 2019). The *luxS* protein homologs that responsible for encoding AI-2 are detected in many bacteria (Liu, Wu et al., 2018). The *luxS* protein can not only regulate the production of AI-2, but also participate in the activated methyl cycle (AMC) (Guo, Gamby et al., 2013). It also can impact the expression of about 400 genes, including those involved in bacterial adhesion and virulence production (Jiang, Chen et al., 2019).

1,8-cineole has shown antibacterial and anti-QS activity against a broad spectrum of pathogenic bacteria. However, the mechanism of 1,8-cineole on influence bacterial biofilm formation, and diminish bacterial virulence is still unknown. In this study, we evaluated the inhibitory effect of 1,8-cineole on the QS-related gene sub minimal inhibitory concentrations, the results showed that 1,8-cineole could inhibit the expression of *luxS* gene, thereafter inhibit the biofilm formation of *Escherichia coli* O101. Further, the *luxS* gene expression inhibitory activity of 1,8-cineole was confirmed by using a new constructed *luxS* gene deletion strain. Finally, the effect of 1,8-cineole on the *luxS* gene was investigated by using an *E. coli* O101 wild-type strain and a *luxS* gene deleted *E. coli* O101 strain infected mouse model.

## Materials and methods

### Bacterial strains, growth conditions and chemicals

*Escherichia coli* O101 was obtained from our laboratory. In a constant temperature shaker at 180 rpm at 37°C, *E. coli* O101 was cultured overnight in Luria–Bertani Broth (LBB, 0.5% yeast extract, 1.0% tryptone, and 1.0% NaCl, at pH 7.0 ± 0.1). The OD_600_ value of *E. coli* O101 was adjusted to 0.4 by Microplate Reader (Bio-Rad, United States), which served as the standard concentration for the following tests. 1,8-cineole (Shanghai Eon Chemical Technology Co., Ltd., Shanghai, China, 99%) was dissolved in 40% acetone and diluted to a final concentration at 50 μl/ml 2% acetone and LB broth were used as controls.

### Determination of minimum inhibitory concentration (MIC) and growth curve

The MIC of 1,8-cineole against *E. coli* O101 was determined using the microdilution method. The experiment was performed with slight modifications according to the clinical and laboratory standards institute (CLSI), United States, 2006. The *E. coli* O101 was diluted to 1 × 10^8^ CFU/ml in LB cultural media and treated with different concentrations of 1,8-cineole (0.19–24.8 μg/ml). The MIC was defined as the minimum concentration of 1,8-cineole that can inhibit the growth of *E. coli* O101.

The growth curve of *E. coli* O101 was used to analyze the inhibitory activity of 1,8-cineole against *E. coli* O101. *E. coli* O101 was grown continuously for 36 h in LB broth containing various concentrations of 1,8-cineole (12.4, 6.2, 3.1, 1.55, 0.78 and 0 μg/ml). 40 μl *E. coli* O101 cultures that had been treated with different concentrations of 1,8-cineole was taken at 0, 1, 2, 4, 8, 12, 16, 24, 36 h, which was uniformly distributed on the LB agar plate to count the colonies after incubating at 37°C for 24 h. For analysis, the number of colonies was transformed to Log10.

### Expression of QS genes and virulence genes on *Escherichia coli* O101

Real-time fluorescence PCR (Bio-Rad, United States) was used to detect the expression of QS and virulence genes in the presence of various doses of 1,8-cineole (3.1, 1.55, 0.78 and 0 μg/ml). Different concentrations of 1,8-cineole were co-cultured with bacteria for 12 h and then total RNA was extracted. TRizol LS Reagent (Biomed, Shanghai, China) was used to extract total bacterial RNA according to the instructions of manufacturer. After that, the total RNA was reverse transcribed according to the instructions of the M-MLV 4 First-Strand cDNA Synthesis Kit (Biomed, Shanghai, China), and real-time PCR was performed in a 10-μl reaction volume. The *rrsG* gene was chosen as the reference gene and used to normalize the quantitative PCR data and calculate the relative differences in mRNA expression using the 2^–△△CT^ method. The primers used to amplify *luxS, lsrK, lsrB, mtn, csgA, csgB, fimE, fimB, rpoS, rrsG* were listed in Table 2.

### *luxS* gene knockout

The upstream and downstream homologous recombination arms of the *luxS* gene internal insertion site were amplified on the *E. coli* O101 genome using ultrafidelity DNA polymerase. Ampicillin (Apr) resistance gene was amplified on plasmid pUC57-Apr. Using fusion PCR, the upstream and downstream homologous recombination arms of *luxS* gene were ligated with the Apr resistance gene to create the entire targeted segment ΔluxS::Apr (Upstream homologous arm-Ampicillin resistance gene-Downstream homologous arm). After then, the targeting fragment was cloned into the suicide plasmid pCVD442 to create the targeting plasmid pCVD442-ΔluxS::Apr. To get donor bacteria β2155/pCVD442-ΔluxS::Apr, pCVD442-ΔluxS::Apr was transferred into *E. coli β2155* using electrotransformation. The donor bacteria β2155/pCVD442-ΔluxS::Apr was spliced with the *E. coli* recipient bacterium and then screened on ampicillin plates to obtain *E. coli* with an ampicillin resistance gene integrated with a targeting plasmid, which was named O101/pCVD442-ΔluxS::Apr. Several O101/pCVD442-ΔluxS::Apr clones were disseminated on LB plates containing 10% sucrose and cultured until a single clone developed. The clone in which the *luxS* gene was replaced by the Apr resistance gene was identified using PCR and named as O101/ΔluxS::Apr.

### Biofilm formation ability

The capacity of *E. coli* O101 to produce biofilms *in vitro* was determined using the 96-well plate crystal violet staining method. The standard *E. coli* O101 and O101/ΔluxS::Apr we added to 96-well plates containing LB broth with various concentrations of 1,8-cineole (3.1, 1.55, 0.78 and 0 μg/ml) respectively to observe whether 1,8-cineole still has a significant inhibitory effect on bacterial biofilm formation without *luxS* gene. Furthermore, the equal concentration of *E. coli* O101 and O101/ΔluxS::Apr were respectively added to 96-well plates containing LB broth. After incubating at 37°C for 24 h, the cultural media was removed and washed with PBS two to three times. The formed biofilm was fixed with 20% formaldehyde for 20 min, and then removed it and dried. Next, staining with 1% crystalline violet for 30 min, the biofilm was washed with PBS and dried. 200 μl 33% glacial acetic acid was added and incubated at 37°C for 2 h to fully dissolve the biofilm. The amount of biofilm was determined by measuring the absorbance value at 595 nm.

### Microscopic analysis of biofilm

The standard *E. coli* O101 and O101/ΔluxS::Apr were respectively placed to a sterile six-well plate containing LB broth with various concentrations of 1,8-cineole (3.1, 1.55, 0.78 and 0 μg/ml). The prepared sterile coverslips were placed in a six-well plate. After incubating at 37°C for 24 h, it was washed with PBS two to three times and fixed with 10% formaldehyde for 20 min. The biofilm was then stained for 30 min with 1% crystal violet to observe the development of biofilm by fluorescence microscope (Nikon, Japan). Silver staining with AgNO_3_ was also utilized to observe the growth of biofilms under the same incubation conditions. The biological boundary structure was further examined using a laser confocal microscope (CLSM). The coverslips containing biofilm were washed with sterile PBS, followed by treatment with different concentrations of 1,8-cineole (3.1, 1.55, 0.78 and 0 μg/ml) for 2 h. DAPI (Solarbio, Beijing) staining solution (10 μg/ml) was added and the slides were stained for 15 min in a light-proof place and observed with CLSM. The maximum excitation wavelength and maximum emission wavelength were 364 nm and 454 nm, respectively. The number of organisms in the biofilm was determined by the fluorescence intensity.

### The motility of *Escherichia coli* O101 and O101/ΔluxS:Apr

The motility of *E. coli* O101 and O101/ΔluxS::Apr were analyzed on agar plates containing different concentrations of 1,8-cineole (3.1, 1.55, 0.78 and 0 μg/ml). *E. coli* O101 was cultured overnight in LB Broth in a constant temperature shaker at 37°C. Then, 10 µl standard *E. coli* O101 was added in the middle of the swarming solid medium (1.0% peptone, 0.5% sodium chloride, 0.5% agar) and swimming soft medium (1.0% peptone, 0.5% sodium chloride, 0.3% agar), respectively. Sterilized toothpicks were dipped in *E. coli* O101 and O101/ΔluxS::Apr respectively and inserted to the bottom of the twitching soft medium (1.0% peptone, 0.5% sodium chloride, 0.1% agar), incubating at 37°C for 24 h. The motility is evaluated by the distance (mm) of the bacteria moving in the culture medium.

### The expression of QS genes and virulence genes on O101/ΔluxS::Apr

To further explore whether the antibacterial mechanism of 1,8-cineole is related to inhibiting the expression of *luxS* gene, real-time fluorescence PCR was used to investigate the expression of QS genes and virulence genes on O101/ΔluxS::Apr. Then, investigated the effect of different concentrations of 1,8-cineole (3.1, 1.55, 0.78 and 0 μg/ml) on the genes expression of O101/ΔluxS::Apr. The bacterial RNA was extracted using TRizol LS Reagent (Biomed, Shanghai, China) according to the instructions of manufacturer and then immediately reverse transcribed according to the M-MLV 4 First-Strand cDNA Synthesis Kit (Biomed, Shanghai, China) instructions. Real-time PCR was performed to test the expression of *lsrK, lsrB, mtn, csgA, csgB, fimE, fimB, rpoS.* The *rrsG* gene was chosen as the reference gene and used to normalize the quantitative PCR data and calculate the relative differences in mRNA expression using the 2^–△△CT^ method. The primers were shown in Table 1.

**TABLE 1:** PCR primers of *Escherichia coli* for analysis of gene expression.

Primers	Sequences (5′→3′)	Tm (°C)
*rrsG-F*	5′-CAT​ACA​AAG​AGA​AGC​GAC​CT-3′	57.1
*rrsG-R*	5′-GAT​TAC​TAG​CGA​TTC​CGA​CT-3′	
*luxS-F*	5′-ACT​TAC​CAG​ATG​CAC​TCG​TTG-3′	58.7
*luxS-R*	5′-AGT​GCC​AGT​TCT​TCG​TTG​CT-3′	
*lsrK-F*	5′-AAC​TTA​AAG​AAC​TGC​ACA​ACA-3′	53.6
*lsrK-R*	5′-ACT​TAA​AGC​CAG​TGT​TTG​TCC-3′	
*lsrB-F*	5′-TCT​GAT​ACT​AAA​CCG​GAG​TGC-3′	58.7
*lsrB-R*	5′-TGG​CTT​TGT​CTT​TAT​TCA​CCT-3′	
*mtn-F*	5′-TGC​CAC​AAT​TTC​AAC​GTC​CC-3′	53.6
*mtn-R*	5′-CAG​CCA​GGA​ACT​CAT​CGA​AG-3′	
*csgA-FcsgA-R*	5′-GTA​ATA​ATA​GCG​GCC​CAA-3′	57.1
	5′-TTA​CCG​CCG​CCA​TGC​TG-3′	
*csgB-F*	5′-CTT​ATG​GTA​ATA​CTG​CGA​TG-3′	58.7
*csgB-R*	5′-GAA​TAG​CCA​TTT​GCG​ACT-3′	
*fimE-F*	5′-ACG​TCG​TTA​TCT​TAC​CG-3′	58.7
*fimE-R*	5′-AAT​CCG​TTC​TTC​AGT​CG-3′	
*fimB-F*	5′-ATA​TTA​TCT​CGA​CTT​CCG​GTG-3′	55.2
*fimB-R*	5′-TAA​GTC​GCG​TAT​CTA​TTC​CC-3′	
*rpoS-F*	5′-ACATCCTGGCCGAT-3′	53.6
*rpoS-R*	5′-AAC​AGC​CAT​TTG​ACG​AT-3′	

### The pathogenicity and therapeutic effect of 1,8-cineole

Kunming mice were randomly divided into six groups (*n* = 6): control group, model group, O101/ΔluxS::Apr model group, 1,8-cineole high-dose group (1.2 g/kg), medium-dose group (0.6 g/kg) and low-dose group (0.3 g/kg), six mice in each group. Except for the normal group, mice in each group were given an intraperitoneal injection with the optimal diarrheal concentration of *E. coli* O101 suspension (0.2 ml/20 g) determined during the pre-experiment. The O101/ΔluxS::Apr model group received the same amount of O101/ΔluxS::Apr suspension as the control group. The normal group was injected with saline. After intraperitoneal injection for 6 h, mice in the 1,8-cineole high-dose group, medium-dose group and low-dose group were gavaged in the morning and evening for 5 days according to their respective dosages, while the remaining groups were gavaged with equal volume of saline, and the body weight of the mice were recorded. At the end of gavage on the fifth day, the blood of mice was collected from the orbital vein, and the amount of different leukocytes in each group of mice was by blood routine test. The concentration of cytokines (IL-1β, IL-6 and TNF-α) in blood was assessed by ELISA (Quanzhou Rui Xin Biotechnology Co., LTD. Fujian, China). Meanwhile, the duodenum of mice in each group was collected for making paraffin sections.

### Statistical analysis

The data were analyzed using parametric statistical tests. Three independent biological replicates were performed for each assay. One-way analysis of variance (ANOVA) was used to analyze differences between the 1,8-cineole, control group and O101/ΔluxS::Apr model group. All data were processed using Statistical Product and Service Solutions (SPSS) 22.0 (IBM Corporation, Armonk, NY, United States) and were presented as mean ± SD. Differences with *p* < 0.05 were considered statistically significant.

## Results

### *In vitro* antibacterial activity of 1,8-cineole

The antibacterial activity of 1,8-cineole was reflected by the MIC value which was evaluated through microdilution method. The results showed that the MIC of 1,8-cineole against *E. coli* O101 was 6.2 μg/ml and MBC was 12.4 μg/ml indicating that 1,8-cineole exhibited an effective anti-bacteria activity against *E. coli*. Comparing *E. coli* O101 growth curves in different concentrations of 1,8-cineole group, it suggested that there was no significant inhibition of bacterial growth when treating with 1,8-cineole at concentrations of 0.78, 1.55 and 3.1 μg/ml (Supplementary Figure S1). Treatment with MIC concentration, bacterial growth was significantly inhibited during the first 4 h, followed by a gradual increase. When the concentration was no more than 1/2 MIC, the growth was slightly inhibited, and then showed a similar trend with untreated bacteria. Therefore, the concentrations of 0.78, 1.55 g and 3.1 μg/ml (1/8MIC, 1/4MIC, 1/2MIC) were chosen to investigate the impact of 1,8-cineole on *E. coli* O101 biofilm and virulence genes.

### The effect of 1,8-cineole on the expression levels of QS genes and virulence genes on *E. coli* O101

**FIGURE 1:**
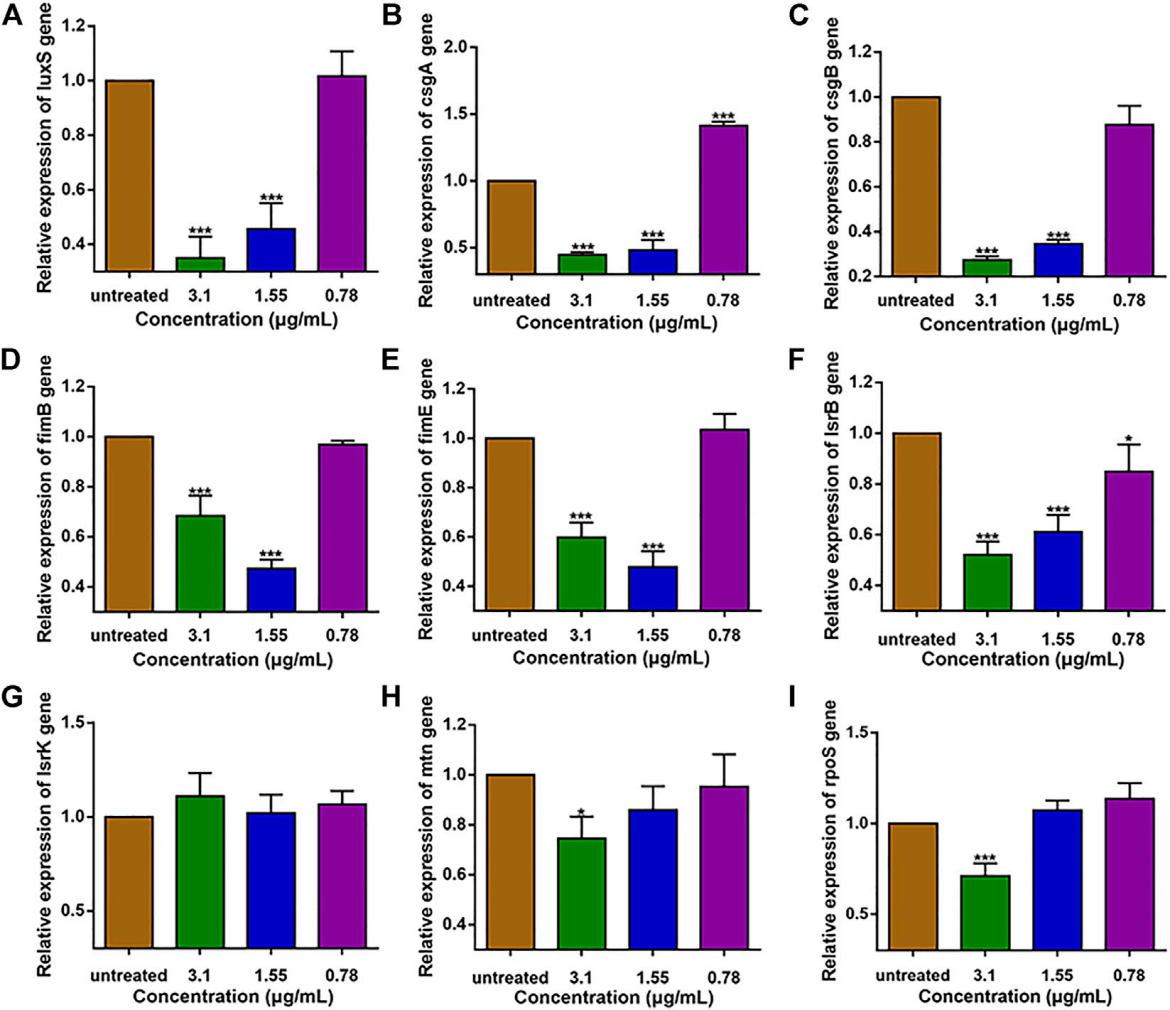
Expression of QS-related and virulence genes in *E. coli* O101 after 18 h inhibition by non-inhibitory 1,8-cineole concentrations. **(A**–**I)**: *luxS, csgA, csgB, fimB, fimE, lsrB, lsrK, mtn, rpoS.* The results represent means ± standard deviations for three independent experiments. *p < 0.05, **p < 0.01 and ***p < 0.001 *versus* the control group.

The expression levels of QS and virulence genes on *E. coli* O101 was determined by qRT-PCR. The results show that 1,8-cineole could effectively suppress (*p* < 0.05) the expression of QS genes and virulence genes, and it exerted best inhibition effect at the concentration of 1/2 MIC (Figure 1). The gene expression inhibition rates of 1/2 MIC 1,8-cineole treatment for 18 h were as follows: *luxS* 65%, *lsrB* 47.9%, *mtn* 25.4%, *csgA* 55.3%, *csgB* 72.4%, *fimB* 31.6%, *fimE* 40.2%, *rpoS* 28.9%. The suppression by 1,8-cineole showed a dose-dependent manner, but the expression of *fimB* and *fimE* genes under the 1/4 MIC concentration of 1,8-cineole was significantly lower than that of 1/2 MIC and 1/8 MIC concentrations. Among the genes regulating QS, the expression of *luxS* gene was most significantly repressed. Therefore, we hypothesized that 1,8-cineole could inhibit the biofilm formation and pathogenicity in *E. coli* O101 *via* suppressing *luxS* gene expression.

### *luxS* gene knockout

The monoclonal colonies that formed on the resistant plates were detected using PCR amplification from the outer side of recombinant arm. The PCR amplification product length of clone No. 6 was changed as predicted (1544 bp for the original strain; 2563 bp for the new constructed strain), indicating that it should be a positive clone with the *luxS* gene interrupted by the insertion of the ampicillin resistance gene. The positive clone was validated by amplification with the outer identifying primers, and the amplification results were submitted for sequencing, which identified that the *luxS* gene had been knocked out (Supplementary Figure S2).

### Biofilm formation ability

**FIGURE 2:**
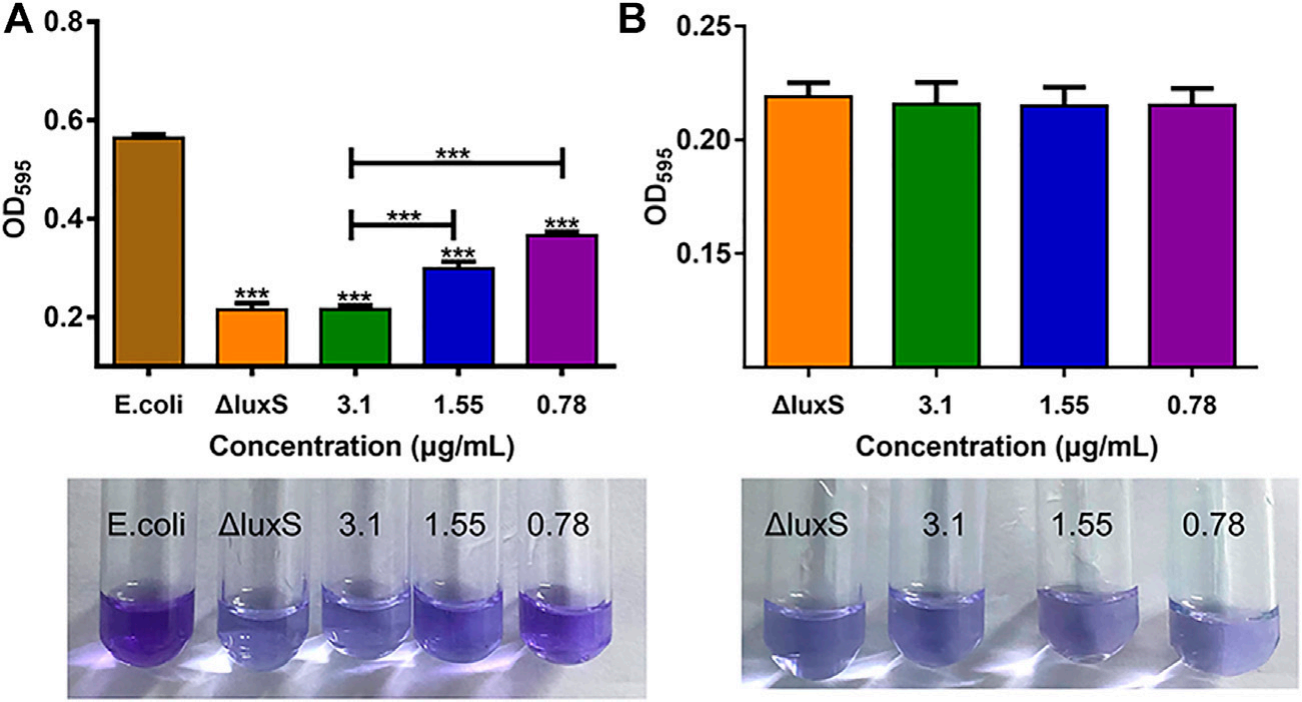
Different influencing factors on the ability of bacterial biofilm formation. **(A)** Effects of *luxS* deletion and 1,8-cineole in non-inhibitory concentrations (3.1, 1.55, 0.78 and 0 μg/ml) on the biofilm formation ability of *E. coli* O101; **(B)** Effect of 1,8-cineole in non-inhibitory concentrations (3.1, 1.55, 0.78 and 0 μg/ml) on biofilm formation of *E. coli* O101 without *luxS* gene. The results represent means ± standard deviations for three independent experiments. *p < 0.05, **p < 0.01 and ***p < 0.001 *versus* the control group.

The impact of 1,8-cineole and *luxS* gene knockout on *E. coli* O101 biofilm formation abilities was shown in Figure 2. The results showed that the suppression rate of biofilm formation by 1,8-cineole at 1/2, 1/4, and 1/8 MIC concentrations was 61.7%, 47% and 35.1%, respectively, indicating that 1,8-cineole has a strong anti-biofilm activity in a dose-dependent manner. The biofilm formation ability of O101/ΔluxS::Apr was dramatically decreased compared to the *E. coli* O101 control group (Figure 2A), with an inhibition rate of 60%, which was comparable to 1/2 MIC concentration of 1,8-cineole (61.7%). Furthermore, 1,8-cineole did not have a significant inhibitory activity on O101/ΔluxS::Apr biofilm formation ability (Figure 2B, *p* > 0.05). These results indicated that the *luxS* gene could regulate the biofilm formation ability of *E. coli* O101 and 1,8-cineole may exert an inhibitory effect on *E. coli* O101 biofilm formation ability by inhibiting *luxS* gene expression.

### Microscopic analysis of biofilm

**FIGURE 3:**
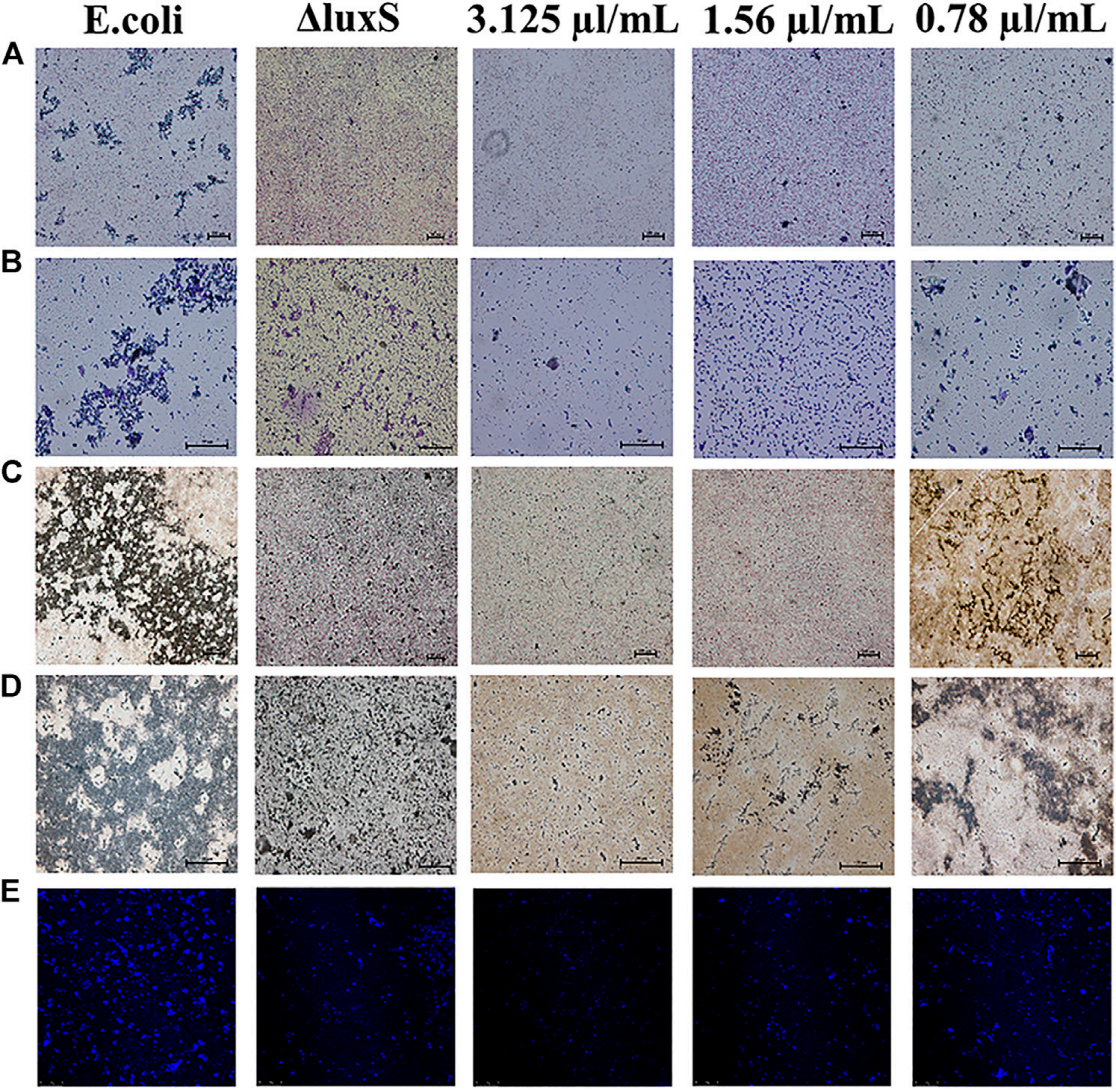
Different influencing factors on the microstructure of bacterial biofilms. **(A**–**E)** Effect of *luxS* gene deletion and non-inhibitory concentrations of 1,8-cineole (3.1, 1.55, 0.78 and 0 μg/ml) on the amount of biofilm formation of *E. coli* O101 under light microscopy and CLSM. **(A**,**B)**: Crystalline violet staining 100× and 400×; **(C**,**D)**: Silver staining 100× and 400×.

**FIGURE 4:**
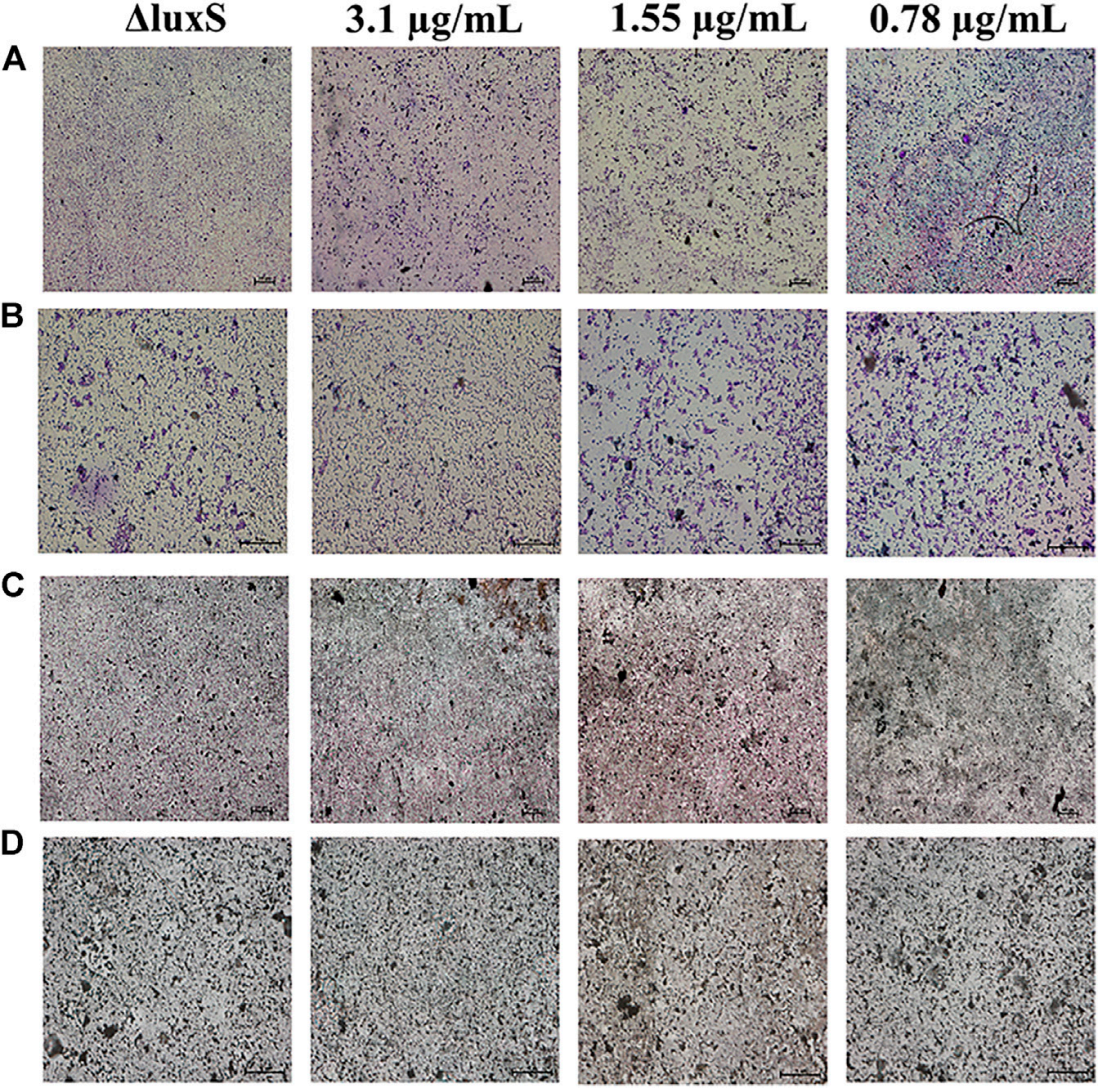
Effect of non-inhibitory concentrations of 1,8-cineole on the microstructure of bacterial biofilms of *E. coli* O101 with deletion of *luxS* gene. **(A**–**D)** Effect of non-inhibitory concentrations of 1,8-cineole on the amount of biofilm formation of *E. coli* O101 without *luxS* gene under light microscopy. **(A**,**B)** Crystalline violet staining: 100× and 400×; **(C**,**D)** Silver staining: 100× and 400×.

The anti-biofilm activity was further investigated using light microscopy and CLSM. The results showed that the biofilm formation was inhibited by 1,8-cineole with a dose-dependent manner in the light microscopy field both stained with crystalline violet or silver. Compared to the control group, the amount of biofilm formation gradually decreased and did not aggregate into sheets as the concentration of 1,8-cineole increased. After the *luxS* gene was deleted, the biofilm formation ability of O101/ΔluxS::Apr was significantly inhibited, which was similar as the results of treating with 1,8-cineole (Figures 3A–D). DAPI staining solution was used to stain live cells or cells that were fixed. 1,8-cineole inhibits bacterial adhesion in a dose-dependent manner, affecting the number of organisms on the crawl and thus the formation of bacterial biofilms. Therefore, we can determine the amount of bacterial cells on the coverslips based on the fluorescence intensity. Under CLSM, it can be observed that as the concentration of 1,8-cineole gradually increased, the fluorescence was gradually decreased, indicating a gradual decrease in the amount of bacterial cells in the biofilm (Figure 3E). Compared with the control group, the fluorescence was significantly reduced after the deletion of *luxS* gene. However, no significant inhibitory effect of 1,8-cineole on O101/ΔluxS::Apr biofilm formation ability was observed within the light microscopic field of view after *luxS* gene deletion (Figure 4). It suggested that the *luxS* gene could regulate the biofilm formation of *E. coli* O101 and 1,8-cineole may exert an inhibitory effect on bacterial biofilm formation by affecting *luxS* gene expression.

### The motility of *Escherichia coli* O101 and O101/ΔluxS::Apr

**FIGURE 5:**
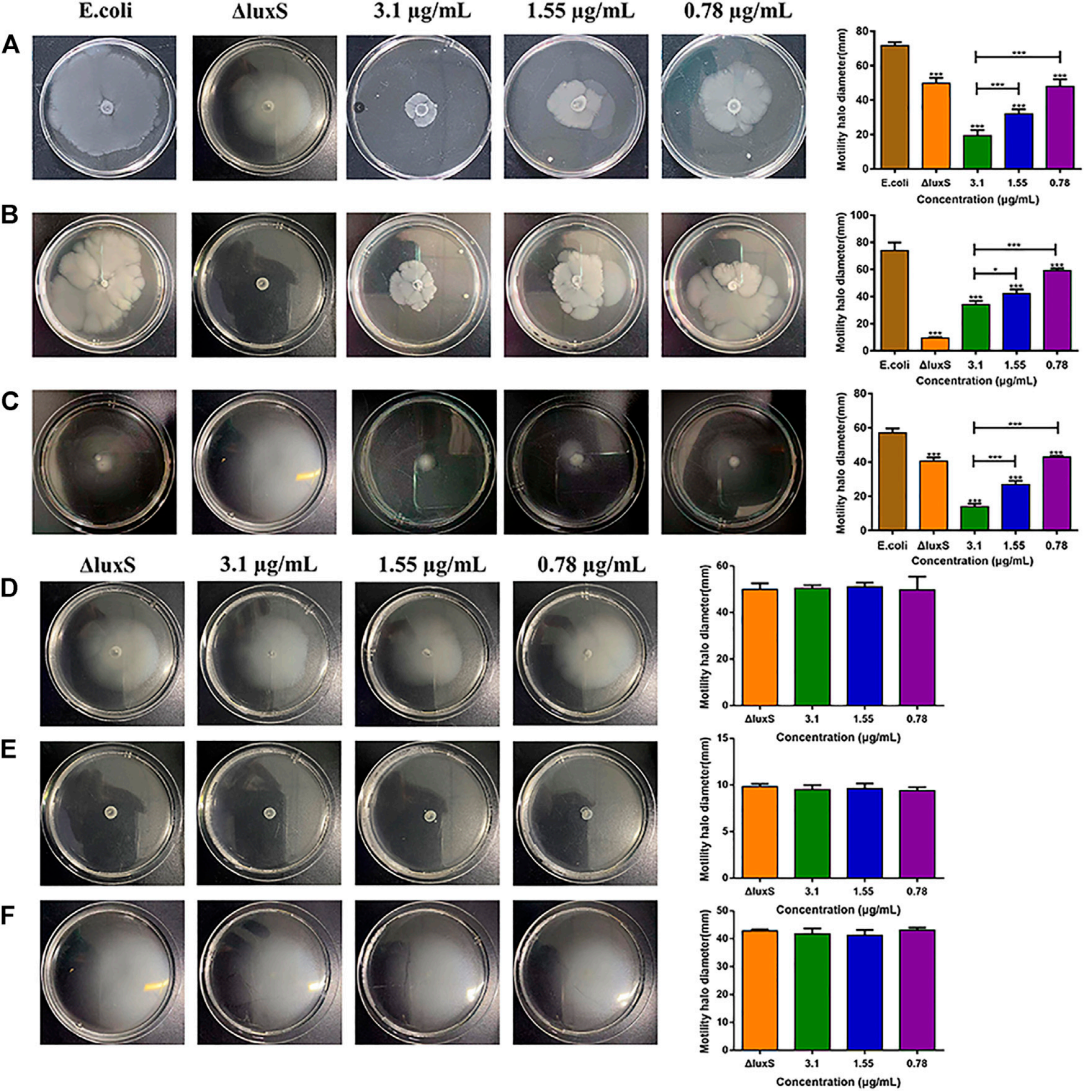
Effect of *luxS* gene deletion and 1,8-cineole on bacterial motility*.* **(A**–**C)** Effect of *luxS* gene deletion and non-inhibitory concentrations of 1,8-cineole on the swimming motility, swarming motility and twitching motility of *E. coli* O101. **(D**–**F)** Effect of non-inhibitory concentrations of 1,8-cineole on the swimming motility, swarming motility and twitching motility of *E. coli* O101 without *luxS* gene. The results represent means ± standard deviations for three independent experiments. *p < 0.05, **p < 0.01 and ***p < 0.001 *versus* the control group.

After treating with 1,8-cineole at doses of 1/2 MIC, 1/4 MIC, and 1/8 MIC, the swimming motility of *E. coli* was decreased by 72.93%, 55.35% and 32.97%, respectively (Figure 5A); the swarming motility decreased by 53.57%, 42.8% and 19.92%, respectively (Figure 5B); the twitching motility decreased by 75.49%, 52.86% and 24.86%, respectively (Figure 5C). Compared with the swimming and twitching motility, the swarming motility of O101/ΔluxS::Apr was inhibited more significantly (*p* < 0.05); In detail, after the deletion of *luxS* gene, the swimming and twitching motility were inhibited by 30.47% and 29.28%, respectively, and the inhibitory effect was comparable to 1,8-cineole at 1/8MIC. However, the swarming motility was inhibited by 87.21%, which was equivalent to the inhibitory effect of 1,8-cineole at 1/2MIC. Meanwhile, the results showed that 1,8-cineole did not have a significant inhibitory effect on the motility of O101/ΔluxS::Apr (Figures 5D–F, *p* > 0.05). For bacterial swimming and twitching motility, 1,8-cineole at concentrations of 1/2 MIC and 1/4 MIC inhibited bacterial motility more significantly compared to *luxS* gene deletion, whereas no significant inhibition was observed for *luxS* gene deletion bacteria under the same treatment conditions. This indicates that the *luxS* gene can regulate other factors that affect bacterial motility. Based on the results of this experiment, we can conclude that *luxS* gene could regulate the motility, but it is not the only regulatory factor and 1,8-cineole exerts its inhibitory effect on bacterial motility by inhibiting *luxS* gene expression.

### The expression of QS-related and virulence genes after *luxS* gene knockout

**FIGURE 6:**
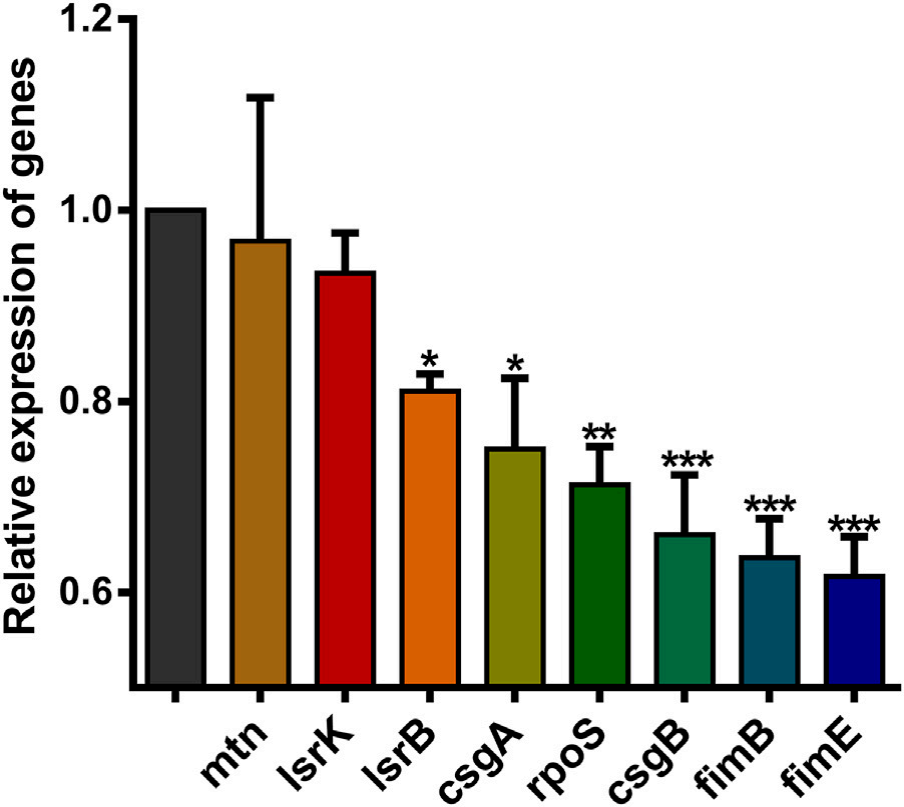
Effect of *luxS* gene deletion on *E. coli* O101 QS-related genes and virulence genes expression. The results represent means ± standard deviations for three independent experiments. *p < 0.05, **p < 0.01 and ***p < 0.001 *versus* the control group.

Real-time fluorescence quantitative PCR was used to investigate the expression level of *E. coli* O101 QS genes and virulence genes. As expected, after the deletion of the *luxS* gene, the mRNA expression levels of related genes were significantly suppressed. The inhibition rates of QS genes and virulence genes were as follows: *lsrB* 19.0%, *lsrK* 6.6%, *mtn* 3.3%, *csgA* 25.0%, *csgB* 34.0%, *fimB* 36.3%, *fimE* 38.4%, *rpoS* 28.7% (Figure 6). In addition, the results showed that 1,8-cineole did not significantly inhibit the expression of QS and virulence genes in O101/ΔluxS::Apr (Supplementary Figure S3), indicating that 1,8-cineole may affect the pathogenicity of *E. coli* O101 by inhibiting the expression of the *luxS* gene.

### The therapeutic effect of 1,8-cineole and the influence of the *luxS* gene on pathogenicity

#### The therapeutic effect of 1,8-cineole against bacterial diarrhea in mice

**FIGURE 7:**
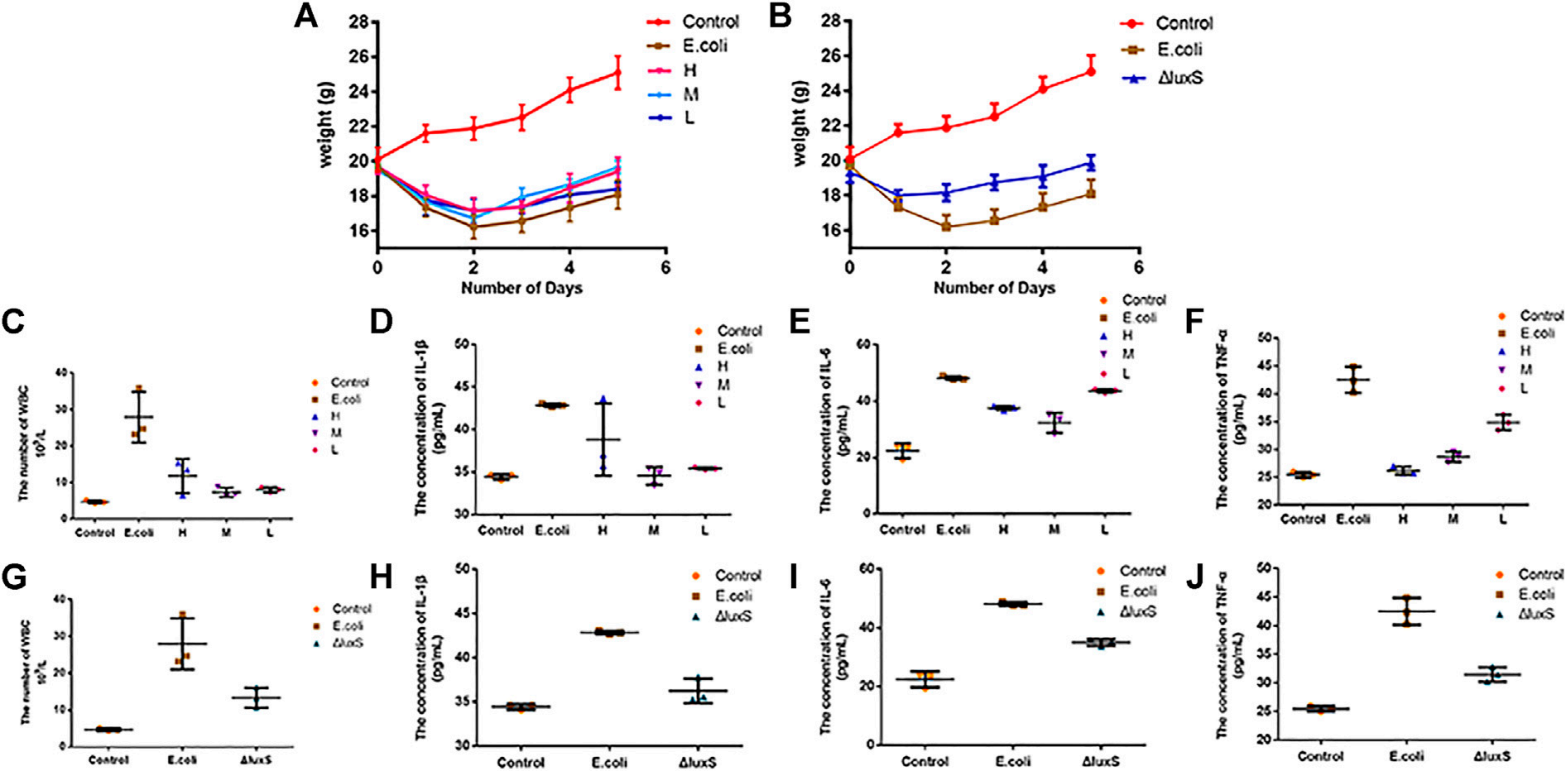
Effect of *Escherichia coli* on body weight and inflammatory factors in mice. **(A)** Body weight changes in mice with bacterial diarrhea after treatment with various dosages of 1,8-cineole. **(B)** The effect of *E. coli* O101 without *luxS* gene on mice body weight. **(C**–**F)** Changes in the concentrations of leukocytes and inflammatory factors (IL-1β, IL-6, and TNF-α) in mice with bacterial diarrhea after treatment with different concentrations of 1,8-cineole. **(G**–**J)** Changes in leukocyte and inflammatory factors concentrations in mice infected with *E. coli* O101 without *luxS* gene. The results represent means ± standard deviations for three independent experiments. *p < 0.05, **p < 0.01 and ***p < 0.001 *versus* the control group.

Animal experiments were performed to investigate the therapeutic effect of 1,8-cineole on bacterial diarrhea in mice, as well as the influence of the *luxS* gene on *E. coli* O101 pathogenicity. The results demonstrated that 1,8-cineole had a good therapeutic effect on *E. coli* O101-induced diarrhea in mice (Figure 7). The mice treated with 1,8-cineole were heavier and gained weight quicker than the model group. Among them, the high-dose group (1.2 g/kg) and the medium-dose group (0.6 g/kg) showed the best treatment effect (Figure 7A) and there was a significant difference in body weight after 5 days compared with the model group (*p* < 0.05). Compared with the model group, the leukocyte levels in the high (1.2 g/kg), medium (0.6 g/kg), and low (0.3 g/kg) dosage groups was decreased by 57.8%, 73.8%, and 71.3%, respectively (Figure 7C). The treatment effect was most significant in the medium dose group, which showed no significant difference with the control group. Compared with the model group, the expression of inflammatory factors IL-1β, IL-6, and TNF-α in mice in the high, medium, and low dosage groups was decreased by 9.4%, 21.9%, 38.4; 19.4%, 32.8%, 32.5%; 17.4%, 9.5%, 18%, respectively (Figures 7D–F). In summary, the inflammatory factor concentration in mice were dramatically inhibited by 1,8-cineole, and the medium-dose group showed the best therapeutic effect.

**FIGURE 8:**
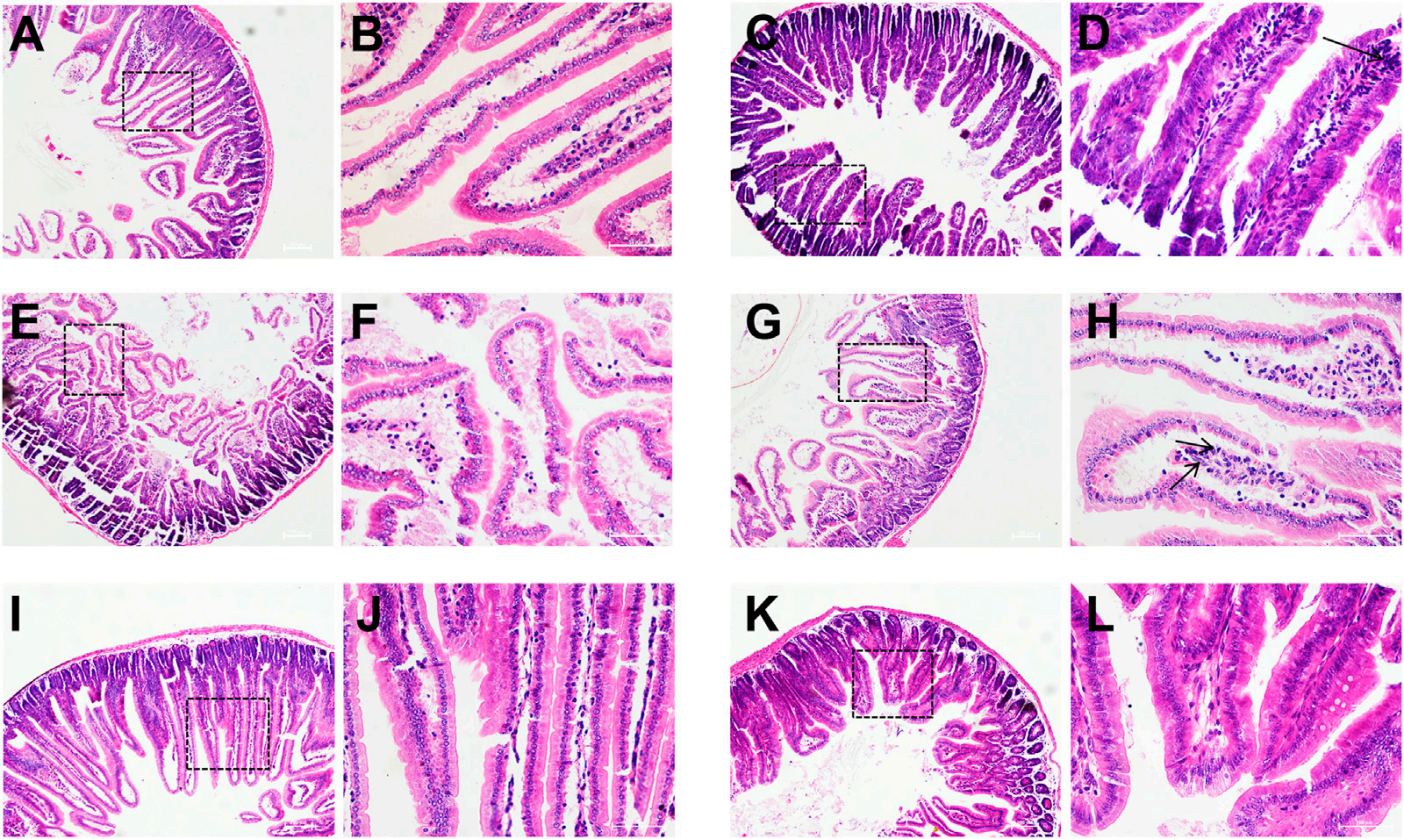
Histopathological observation of mice duodenum. Control group **(A**,**B)**, model group **(C**,**D)**, O101/ΔluxS::Apr model group **(E**,**F)**, 1,8-cineole low-dose group **(G**,**H)** (0.3 g/kg), medium-dose group **(I**,**J)** (0.6 g/kg) and high-dose group **(K**,**L)** (1.2 g/kg). **(A**,**C**,**E**,**G**,**I**,**K)**, HE, 100×; **(B**,**D**,**F**,**H**,**J**,**L)**, HE, 400×.

Compared with the control group, a large number of inflammatory cells infiltrating within the field of view (neutrophils, lymphocytes, macrophages, *etc.*) in the model group. Intestinal glands were disorganized and intestinal villi were atrophied and broken, and intestinal structures were severely damaged. Compared with the model group, there was no inflammatory cell infiltration within the field of view and the structure of the intestinal villi was intact in the high-dose group. In the low-dose group, the intestinal structure was relatively intact, but there were still a few inflammatory cells infiltrated in the intestinal villi (Figures 8A,B,D–F).

#### The effect of *luxS* gene deletion on the pathogenicity of *E. coli* O101

The pathogenicity of *E. coli* O101 in mice was greatly decreased by knocking out the *luxS* gene. The mice in the O101/ΔluxS::Apr model group were healthier than the model group in terms of both mental health and diarrhea severity. After 5 days, there was a significant difference in body weight compared to the model group of mice (*p* < 0.05), and the weight of mice in the O101/ΔluxS::Apr model group was significantly higher than that in the model group (Figure 7B). Compared with the model group, the concentrations of leukocytes, inflammatory factors IL-1β, IL-6, and TNF-α *in vivo* was decreased by 52.3%, 15.5%, 27.2%, 25.9%, respectively (Figures 7G–J). The Paraffin section showed that the deletion of *luxS* gene resulted in a significant reduction in the ability for *E. coli* O101 damaging the intestine. Compared with the model group, inflammatory cells were significantly reduced within the field of view, and the breakage of intestinal villi was not significant (Figure 8C).

## Discussion

During the regulation of quorum sensing in bacteria, a series of diffusible and secreted signals are involved in it. These signals can vary widely between different types of bacteria (Abisado, Benomar et al., 2018), which have shown significant advantages as antibacterial targets in antibacterial research in recent years. Quorum-sensing-mediated inter-bacterial communication has been recognized as an important signaling communication mode in bacterial flora (Mukherjee and Bassler 2019b). Numerous studies have shown that bicyclic terpenoids have significant antibacterial and anti-biofilm activities (Sharifi, Mohammadzadeh et al., 2021). 1,8-cineole is the active ingredient in the essential oil extracted from most camphoraceous plants. Previous researches have revealed that as a terpenoid, it has strong antibacterial activity (Kifer, Mužinić et al., 2016; Merghni, Noumi et al., 2018). In this study, the antibacterial activity of 1,8-cineole showed that its MIC against *E. coli* O101 was 6.2 μg/ml, which had a strong inhibitory effect on bacterial growth, but the bacterial growth was not suppressed at doses ranging from 0.78 to 3.1 μg/ml. After treatment with 2MIC 1,8-cineole, the bacteria were completely killed within 2 h, showing a significant bactericidal activity. Compared with commonly used antibiotics (gentamicin, amoxicillin/clavulanic acid, *etc.*), 1,8-cineole exhibited weaker bactericidal activity, but when antibiotics were combined with 1,8-cineole, the MIC of antibiotics could be significantly reduced, achieving the purpose of reducing the use of antibiotics (Hriouech, Akhmouch et al., 2020; Mączka, Duda-Madej et al., 2021). The formation of *E. coli* biofilms can be regulated by the small molecule autoinducer-2 (AI-2), a product of the *luxS* enzyme. *LuxS* is part of the activated methyl cycle, which can also affect biofilm development by AI-2-independent effects on metabolism (Niu, Robbins et al., 2013). Many studies had shown that the *luxS* gene has a significant effect on the biofilm forming ability and pathogenicity of bacteria (Yoshida, Ansai et al., 2005; Yadav, Vidal et al., 2018). The anti-QS activity of 1,8-cineole has been confirmed by previous studies, but it has not been reported that whether 1,8-cineole is primarily targeted with *luxS* gene to interfere with the QS system. In this study, we have systematically studied the inhibitory effect of 1,8-cineole (sub-MICs) on the QS system of *E. coli* from the QS phenotype level.

*Pfs* (*mtn*) is an AI-2 synthase that can regulate the synthesis of AI-2 (Zhang, Qi et al., 2017). *LsrK* is one of the key components of the *luxS*-regulated (lsr) operon in *Escherichia coli* and plays an important role during the quorum-sensing (QS) process mediated by autoinducer-2 (AI-2) (Ha, Eo et al., 2017). *LsrB* regulates AI-2 transport in the bacterial QS system. *RpoS* regulates the expression of a number of genes involved in the external environment as well as genes involved in biofilm formation. Furthermore, the high pathogenicity of *E. coli* is closely related to multiple virulence factors, including adhesion, iron-binding, proteins, antiserum factors, outer membrane proteins, and hemolysis, such as *fliC*, *fimA* and *fimB* (type 1 pili), *csgA* and *csgB* (curly pili A), and so on (Peng, Yuan et al., 2019). The qRT-PCR results revealed that at non-inhibitory doses, 1,8-cineole had a substantial inhibitory impact on *E. coli* O101 QS and virulence genes, and among the genes regulating QS, the most significant inhibition was observed in the *luxS* gene. The deletion of *luxS* gene could significantly affect the expression of virulence genes, which had important implications for bacterial pathogenicity (Lyon, Madden et al., 2001; Jones, Peterson et al., 2010). In addition, *csgA* gene expression was significantly higher in the presence of 1/8 MIC 1,8-cineole when compared to the control group. The results suggested that bacteria could selectively regulate gene expression to enhance self-defense mechanisms (e.g., flagella) when they encountered adverse factors in the environment.

Biofilms are one of the important causes of bacterial resistance and virulence (Del Pozo 2018), and they are significantly less susceptible to antimicrobial agents compared to non-adherent cells. Biofilm-based infections are difficult to treat as a result of this (Caputo et al.). Therefore, biofilm-based infections are difficult to treat and inhibition of biofilm formation is a viable method to attenuate the virulence and drug resistance of disease pathogens (Packiavathy, Priya et al., 2014). The non-inhibitory concentration of 1,8-cineole inhibited biofilm formation by 61.7%, while the biofilm formation ability decreased by 60% after *luxS* gene deletion, and the inhibitory effect was comparable to that of 1,8-cineole. Bacterial motility is associated with virulence factors such as fimbriae and flagella, which contribute to bacterial pathogenicity (Mannan, Rafique et al., 2020). The inhibition of the bacterial fimbriae could have a significant impact on its invasive ability, which was previously demonstrated in *Salmonella* (Xiong, Liu et al., 2020). Swarming involves the differentiation of vegetative cells into hyper-flagellated swarm cells that undergo rapid and coordinated population migration across solid surfaces, and swimming and swarming behavior essentially determine the biofilm formation (Wang, Huang et al., 2019). The results showed that all three forms of motility were inhibited by treatment with 1,8-cineole and in the absence of the *luxS* gene. Previous studies have shown that the motility is required early perhaps to locate a suitable environment or surface on which to form a biofilm, but bacterial motility receives significant inhibition once the biofilm is formed (Guttenplan and Kearns 2013). The non-inhibitory concentration of 1,8-cineole and the absence of the *luxS* gene not only inhibited bacterial biofilm formation, but also inhibited bacterial motility, which resulted in a great reduction of bacterial pathogenicity. These results suggested that both bacterial biofilm and motility could be the targets of 1,8-cineole, and the *luxS* gene is the main gene that regulates bacterial biofilm formation and motility. However, 1,8-cineole did not significantly inhibit the motility of *luxS* gene-deleted *E. coli*. Meanwhile, the qRT-PCR results revealed that 1,8-cineole did not have a substantial inhibitory impact on the QS and virulence genes of *E. coli* with *luxS* gene deletion at non-inhibitory doses, indicating that 1,8-cineole can affect the QS and biofilm formation through inhibiting *luxS* gene expression. The antibacterial mechanism of 1,8-cineole is not representative of all terpenoids, and the antibacterial targets of terpenoids are often different. Proteins can also be used as antimicrobial targets of terpenoids (Sj, Ck et al., 2020). The essential oil from *Melaleuca alternifolia* is used as an antibacterial, and molecular docking indicated that penicillin-binding protein 2a of *S. aureus* is a possible target of terpinen-4-ol which is main constitute of the oil (Cordeiro, Figueiredo et al., 2020).

Previous studies have demonstrated that the *luxS* gene could regulate bacterial pathogenicity. The deletion of *luxS* gene in *Histophilus somni* significantly affected the pathogenicity of the bacteria in mice (Pan, Siddaramappa et al., 2021). The 50% lethal dose (LD50) of the *E. piscicida* ΔluxS strain for zebrafish was significantly higher than that of the wild-type strain, which suggests that the *luxS* gene deletion could attenuate virulence of *E. piscicida* (Sun, Li et al., 2020). *Campylobacter jejuni* completely lost the ability to colonize the gut due to deletion of the *luxS* gene. The mutant also failed to induce miscarriage in a pregnancy model, whereas the wild-type strain had a high rate of miscarriage (Plummer, Sahin et al., 2012). In animal experiments, 1,8-cineole exhibited good therapeutic effects, and the deletion of *luxS* gene significantly reduced effects on mouse body weight. Leukocytes circulate throughout the body of animal *via* blood and lymphatic vessels. During circulation, they can sense changes in the vascular microenvironment and participate in the immune response (Lee, Breuer et al., 2021). Compared with the model group, the number of leukocytes in mice treated with 1,8-cineole decreased by 56.1%, and the number of leukocytes in mice with *luxS* gene deletion decreased by 52.3%. The cytokine interleukin-1β (IL-1β) is an important mediator of inflammatory responses. It is necessary for host responses and pathogen resistance, but it also exacerbates damage during chronic disease and acute tissue injury (Lopez-Castejon and Brough 2011). Interleukin 6 (IL-6) acts as an immune defense by stimulating acute phase responses, hematopoiesis, and immune responses, which can produce rapidly and transiently upon infection and tissue damage (Tanaka, Narazaki et al., 2014). TNF-α is an important pro-inflammatory cytokine with strong pro-inflammatory activity and can promote the secretion of various pro-inflammatory mediators (Wang, Che et al., 2020). The ELISA assay results of these three inflammatory factors showed that 1,8-cineole can effectively alleviate inflammation in mice. The pathogenicity was significantly reduced after deletion *luxS* gene, resulting in a significant reduction in the inflammatory response, and the inhibition rates of inflammatory factors were 15.5%, 27.2% and 25.9%, respectively.

## Conclusion

Here we show that, 1,8-cineole has substantial antibacterial and anti-biofilm action, notably, it has significant inhibition activity on the *luxS* gene. 1,8-cineole could inhibit biofilm formation and reduce pathogenicity of pathogenic bacteria by inhibiting the expression of *luxS* gene. These results demonstrate that 1,8-cineole is a quorum sensing inhibitor, which is a promising candidate for the development of novel and effective antibacterial.

## Data Availability

The original contributions presented in the study are included in the article/Supplementary Material, further inquiries can be directed to the corresponding author.
