# Supplementary material for: 1,8-Cineole inhibits biofilm formation and bacterial pathogenicity by suppressing luxS gene expression in Escherichia coli
Source: Front Pharmacol. 2022 Oct 14;13:988245. doi: 10.3389/fphar.2022.988245 (PMC9624193; doi:10.3389/fphar.2022.988245)
Supplement: Supplementary file 1 [file DataSheet1.doc]

**Supplementary Material**

**1,8-Cineole Inhibits Biofilm Formation and Bacterial Pathogenicity by Suppressing *luxS* gene Expression in *Escherichia coli***

Yiming Wang^1†^, Yu Zhang^1†^, Xu Song^1†^, Chunlin Fang ^2，3^, Rui xing^1^, Lu Liu^1^, Xinghong Zhao^1^, Yuanfeng Zou^1^, Lixia Li^1^, Renyong Jia^4^, Gang Ye^1^, Fei Shi^1^, Xun Zhou^1^, Yingying Zhang^1^, Hongping Wan^4^, Zhongqiong Yin^1*^

^1^ Natural Medicine Research Center, College of Veterinary Medicine, Sichuan Agricultural University, Chengdu, China

^2^ Chengdu Agricultural College, Chengdu, China.

^3^ Chengdu QianKun Veterinary Pharmaceutical Co., Ltd, Chengdu, China.

^4^ Key Laboratory of Animal Disease and Human Health of Sichuan Province, Sichuan Agricultural University, Chengdu, China*

†Co-first author


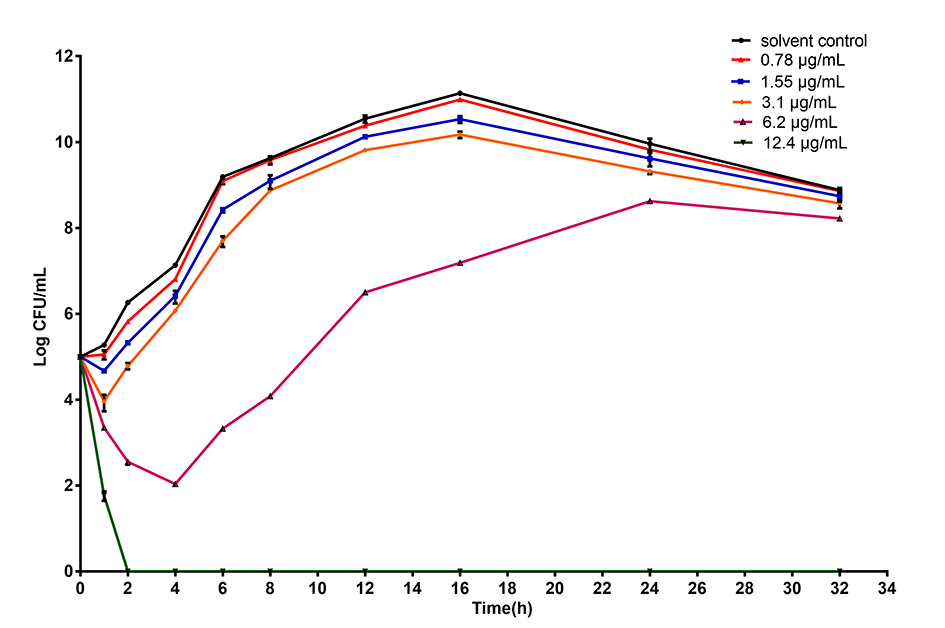


**Supplementary Figure S1|** **Growth curves of *E. coli* O101 under the treatment of different concentrations of 1,8-Cineole** (12.5, 6.25, 3.125, 1.56, 0.78, and 0 μL/mL). The results represent means ± standard deviations for three independent experiments.


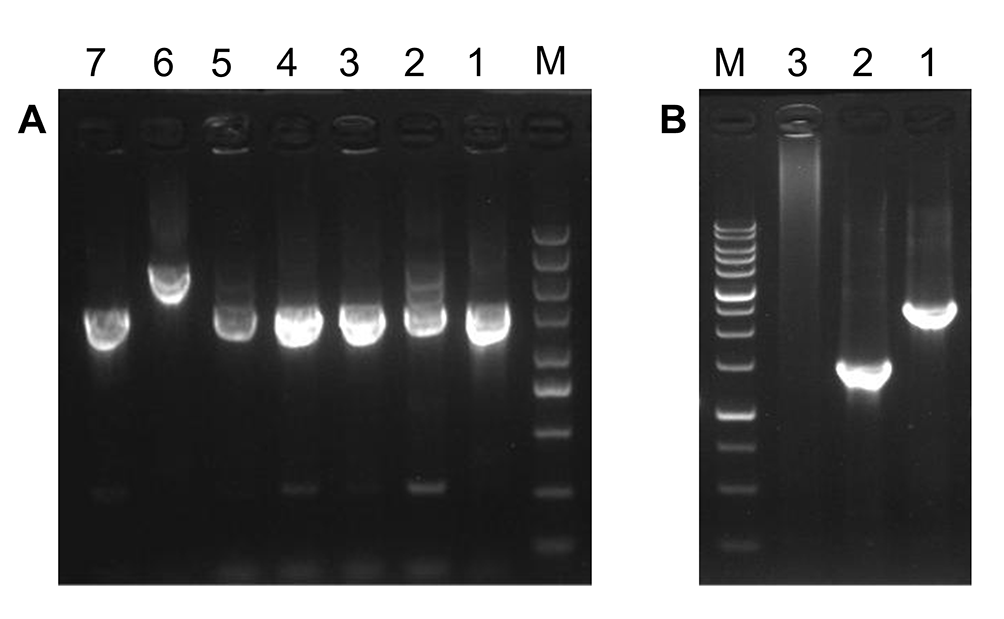


**Supplementary Figure S2|** **Screening and identification of *luxS* knockout bacteria.** A, B: Electropherogram for *luxS* gene knockout monoclonal bacteria screening and identification. In figure A, M: DNA marker, 1-6: the results of amplification of the outer primers. 7: the result of amplification of the original strain. In figure B, M: DNA marker, 1: Amplification results of the outer primers of the overnight culture broth. 2: the result of amplification of the original strain. 3: Amplification results without template negative control.


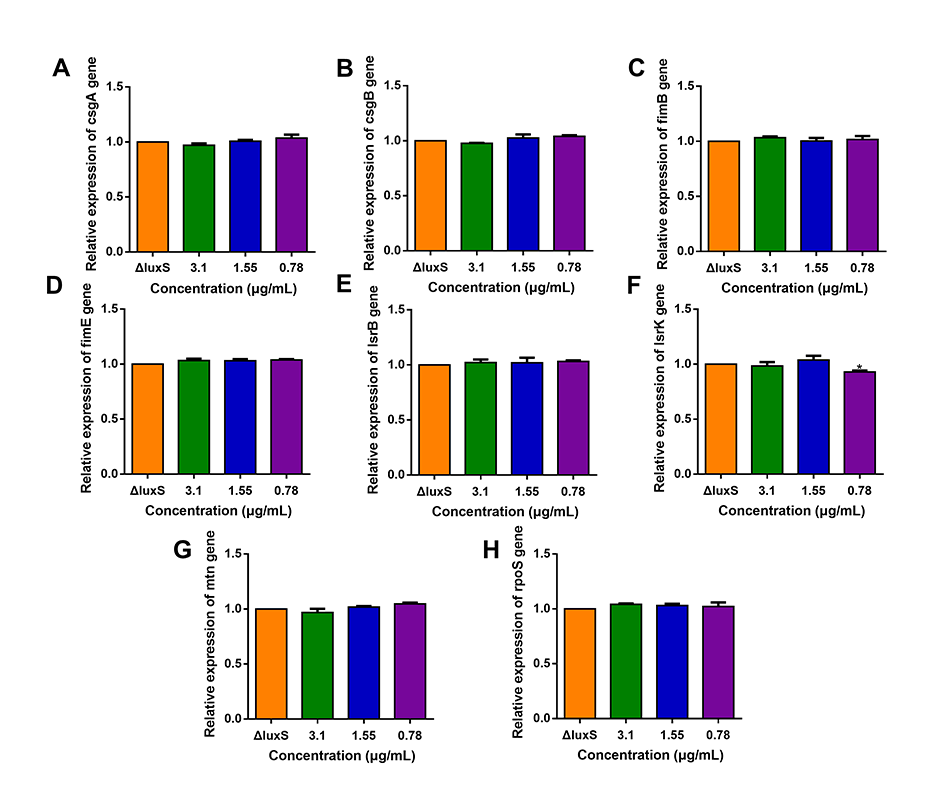


**Supplementary Figure S3|** **Effect of non-inhibitory concentrations of 1,8-cineole on QS-related genes and virulence genes expression of *E. coli* O101 without *luxS* gene.** The results represent means ± standard deviations for three independent experiments. *p < 0.05, **p < 0.01 and ***p < 0.001 versus the control group.
